# SumStatsRehab: an efficient algorithm for GWAS summary statistics assessment and restoration

**DOI:** 10.1186/s12859-022-04920-7

**Published:** 2022-10-25

**Authors:** Mykyta Matushyn, Madhuchanda Bose, Abdallah Amr Mahmoud, Lewis Cuthbertson, Carlos Tello, Karatuğ Ozan Bircan, Andrew Terpolovsky, Varuna Bamunusinghe, Umar Khan, Biljana Novković, Manfred G. Grabherr, Puya G. Yazdi

**Affiliations:** SelfDecode.Com, 1031 Ives Dairy Road Suite 228 - 1047, Miami, FL 33179 USA

**Keywords:** Bioinformatics, GWAS, Summary statistics, PRS, Genetics

## Abstract

**Background:**

Generating polygenic risk scores for diseases and complex traits requires high quality GWAS summary statistic files. Often, these files can be difficult to acquire either as a result of unshared or incomplete data. To date, bioinformatics tools which focus on restoring missing columns containing identification and association data are limited, which has the potential to increase the number of usable GWAS summary statistics files.

**Results:**

SumStatsRehab was able to restore rsID, effect/other alleles, chromosome, base pair position, effect allele frequencies, beta, standard error, and *p*-values to a better extent than any other currently available tool, with minimal loss.

**Conclusions:**

SumStatsRehab offers a unique tool utilizing both functional programming and pipeline-like architecture, allowing users to generate accurate data restorations for incomplete summary statistics files. This in turn, increases the number of usable GWAS summary statistics files, which may be invaluable for less researched health traits.

## Background

A major goal of modern precision medicine is to accurately predict individual health risks based on genetic data [1]. Alongside the advent of next-generation sequencing (NGS) technologies has come a plethora of discoveries linked to genome-wide association studies (GWAS) [2]. The large amount of data generated by these studies has enabled researchers to apply statistical techniques in order to generate polygenic risk scores (PRS) [3].

These scores can be used to predict an individual's genetic risk of a particular health condition. An example of a method that can generate PRS is PUMAS. PUMAS uses trait-specific GWAS summary statistics files for training, in order to fine-tune its predictive model [4]. The core limitation of these techniques is the availability of high-quality GWAS summary statistics.

Summary statistics are used to convey key GWAS data such as variant ID (rsID), chromosome number (Chr), base pair position (BP), effect allele (EA), other allele (OA), minor allele frequency (MAF), t-statistics, *p*-value and standard error (StdErr). However, summary statistics from GWAS are often not shared, and there is no universally standardized format, even with regards to what data is reported and what is not [5–7]. GWAS summary statistic files are also often presented in a multitude of tabular formats, including plink, CTA, BOLT-LMM, GEMMA, Matrix eQTL, METAL, and VCF [6, 7]. As a result, some of the information needed for meta-analysis or downstream GWAS applications—rsID, Chr, BP, OA, EA, MAF, StdErr, Beta, *p*-value—may be missing from the files [6]. Additionally, variation in methodologies of genotyping arrays and quality control filters used by different research groups may contribute to missing SNP identification or association data [8].

Missing columns of data may influence the predictive power of techniques used to generate a PRS, or even render the GWAS file unusable. This can be a particular problem if there are a limited number of high-power studies for a particular trait of interest, which is often the case with the majority of GWAS publications not publicly sharing their data [5]. For these files, restoration would be particularly prudent.

To date, there are no tools which restore this data perfectly. Murphy et al. recently developed MungeSumstats, an R software package, which manually standardizes and performs quality control on different GWAS summary statistic files [7]. This tool performs several quality control steps in order to ensure all key data is present and consistently formatted. Part of this quality control includes restoration of some incomplete data columns, though this is not the main function of the tool. Additionally, the quality of restoring rsID using MungeSumstats is limited by the most recently curated version of the SNPlocs database [9]. To our knowledge, no tool currently attempts to restore missing standard error, Beta, or *p*-values.

In order to address these issues, we developed SumStatsRehab. This tool is able to perform restoration of rsID, chromosome, base pair position, effect allele frequencies, back-calculation of t-statistics from *p*-values, beta value restoration, and standard error calculations and corrections. Once restored, the output is presented in a consistent tabular form. Additionally, SumStatsRehab can diagnose cases where critical data cannot be restored in a given GWAS summary statistics file, and can thus be used for both quality control and cleanup of files. In this paper, we describe SumStatsRehab, its features and utility. We also provide a comparison with the only current alternative for summary statistics restoration, MungeSumstats. The source code for SumStatsRehab is found at https://github.com/Kukuster/SumStatsRehab.

## Methods

### Implementation

**Fig. 1:**
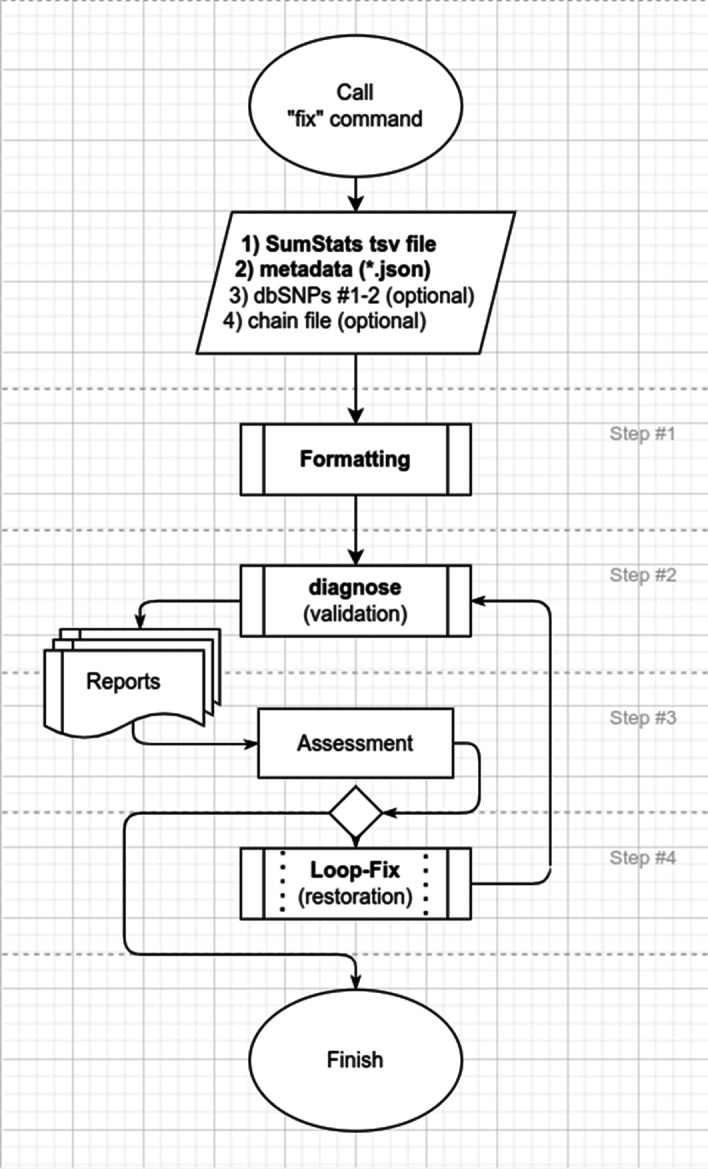
SumStatsRehab implementation pipeline

SumStatsRehab is written in Python3, and utilizes several native Linux executables. The key functions of SumStatsRehab are assessment, validation and restoration (Fig. 1; Table 1). These functions can be implemented for chromosome, base pair position, rsID, effect allele, other allele, allele frequency, standard error, beta, and *p*-value. Each category of data in the input GWAS summary statistic file is assessed, validated, and restored independently. SumStatsRehab accepts GWAS summary statistic files with single nucleotide polymorphisms (SNPs) which reference human genome build 36, build 37, and build 38, and can output restored summary statistics files in reference builds 37 and 38. SumStatsRehab uses a.json header file to correctly read and interpret the columns in the input summary statistic file.

**Table 1:** Comprehensive list of the steps implemented by SumStatsRehab

Step #	Description	Stage
1	Check if the input file is a zip or a gzip archive. If so, extract the raw file	Formatting
2	Pick and reorder the columns to the internal format, based on the corresponding *.json config file	
3	Cut the ‘chr’ prefix in the chromosome column, if present	
4	Calculated the weighted average of EAF columns into one EAF column, if multiple were specified	
5	Check if a chromosome entry is an integer from 1 to 23, or X, Y, M. If an entry is not, mark it as invalid	Validation
6	Check if a base pair position entry is a non-negative integer. If an entry is not, mark it as invalid	
7	Check if an rsID entry is a non-negative integer with ‘r’ prefix. If an entry is not, mark it as invalid	
8	Check if an effect allele or other allele entry is either a dash or composed of letters ATCG. If an entry is not, mark as invalid	
9	Check if a *p*-value or an EAF entry is a real value between 0 and 1 inclusively. If an entry is not, mark as invalid	
10	Check if a standard error or a beta entry is a real value. If an entry is not, mark as invalid	
11	Calculate statistics and save the report about correctness of the data	
12	Analyze the report, If no resolvable issues were found, finish the execution	Analysis and preparation
13	If resolvable issues were found, prepare the restoration algorithm depending on the issues	
14	Perform the liftover to build 38 if needed	
15	Sort the sumstats file either by ChrBP or by rsID, depending on the restoration algorithm	
16	If a chromosome or a BP entry was marked as invalid, and the sumstats file is sorted by rsID, then restore both entries by a lookup in the dbSNP for matching rsID	Restoration
17	If an rsID entry was marked as invalid, and the sumstats file is sorted by ChrBP, then restore rsID entry by a lookup in the dbSNP for matching Chr and BP	
18	If from EA and OA entries only one is invalid then restore the invalid allele as the most likely allele from known by a lookup in the dbSNP either for matching rsID and valid allele, if sumstats is sorted by rsID, or for matching Chr, BP, and valid allele, if sumstats is sorted by ChrBP	
19	If an EAF entry was marked as invalid, then EAF is restored by a lookup in the dbSNP either for matching rsID and effect allele, if sumstats is sorted by rsID, or for a matching Chr, BP, and effect allele, if sumstats is sorted by ChrBP	
20	If a standard error, beta, or *p*-value entry was marked as invalid, and the other two entries as valid, then restore the invalid using the formula s = β/z, where s is the standard error, β is beta, and z is z-score that corresponds to the *p*-value in the two-tailed test [10]	
21	Go back to step #5 (Validation stage)	

### Assessment and validation of summary statistics files

**Fig. 2:**
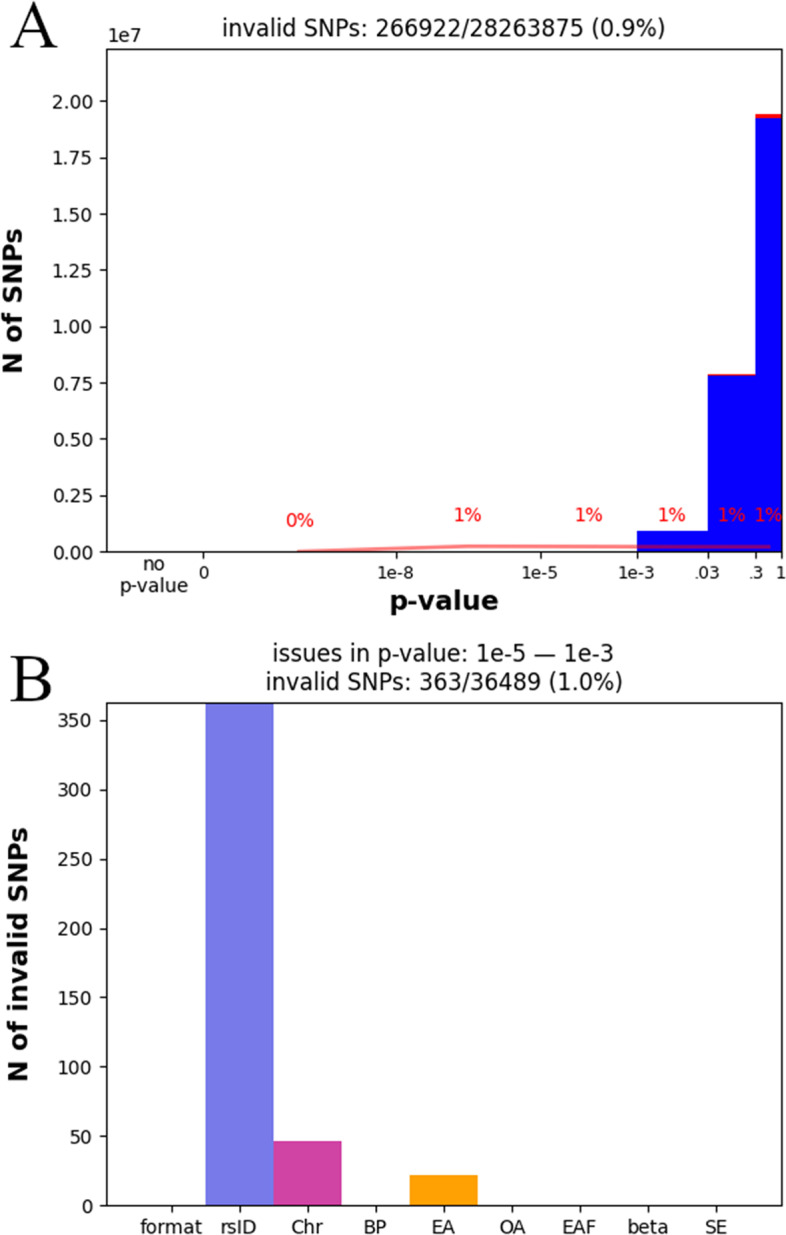
**A** stacked histogram plot—the core plot produced by the “diagnosis” command. It maps all SNPs against their *p*-value, with the valid portion of SNPs colored blue and invalid SNPs colored red, allowing assessment of the distribution of invalid SNPs by significance. **B** One of several bar charts produced by the “diagnosis” command. This plot is generated for each bin of the stacked histogram plot **A** and reports the number of issues that invalidate the SNP entries in a particular bin

SumStatsRehab can be used to identify any invalid SNPs in a GWAS summary statistic file (“fix” command in Fig. 1); invalid SNPs are those which are missing any of the key GWAS data listed in the Background section. This enables users to determine the number and cause of missing or invalid SNPs (Fig. 2).

To demonstrate this command, we tested it on an example GWAS summary statistics file (GWAS blood pressure [11]). As shown, SumStatsRehab identified that less than 1% of entries for GW-significant SNPs were missing Fig. 2A), and that the majority of missing entries were rsIDs (Fig. 2B). The resultant plots derived using the diagnostic tools assess the number of invalid SNPs by significance level, showing the potential impact of the incomplete data columns on downstream calculations. The results of this diagnostic are used internally to guide and optimize restoration.

### Restoration of summary statistics files

SumStatsRehab only attempts to restore entries identified as invalid, with one exception. When either the base pair position or chromosome is invalid, SumStatsRehab restores both by looking up the rsID associated with that entry, and overwriting the chr and base pair position entries.

The extent of restoration possible is dependent on the inputs to SumStatsRehab. If only the summary statistics file is provided as input, SumStatsRehab will be able to perform restoration of the *p*-values, betas and standard errors given two out of three of these values are present. The additional input of a dbSNP file in the target human genome reference build is optimal for restoration. SumStatsRehab preprocesses the dbSNP file, organizing it by rsID, chromosome, base pair position, alleles, ref/alt, and frequencies associated with each SNP, sorted by chromosome and base pair position, and by rsID. If the target build and the GWAS summary statistic file builds are different, an additional third input, the ‘chain file’ is needed for liftover from the summary statistic file build to the target build. With these inputs, SumStatsRehab is able to restore GWAS data files, and include effect allele frequencies (EAF), missing t-statistics, rsID or chromosome numbers and base pair positions, effect allele (EA) and other allele (OA).

### Preparation of test case files

To assess the utility of our tool and the extent of restoration it can achieve, we chose publicly available and complete summary statistics files from three different GWAS as test cases: (1) blood pressure, (2) C-reactive protein, (3) allergies [11–13]. These files were preprocessed by removing one specific column of data per file at a time: rsID, chromosome number, base pair position, effect or other allele, allele frequency, *p*-value, beta, and standard error. After removing each column from the three different GWAS summary statistics files to generate a total of 9 test files per GWAS, with a total of 27 test files, we ran each file through SumStatsRehab using dbSNP versions 144 and 155 for comparison, as well as the only current alternative, MungeSumstats. Version 144 of dbSNP was chosen as it matched the same build utilized by MungeSumstats due to this being the most up to date SNPlocs database; dbSNP155 was chosen due to it being the most up to date dbSNP database at the time the work was carried out.

In order to be run through MungeStumstats, test files required an extra round of extensive preprocessing. For the blood pressure test files, all columns were renamed in accordance with the MungeSumstats documentation [7]. For the GWAS allergies test files, all fields containing ‘NA’ had to be replaced with a placeholder dot, and all rows with any non-numeric value in BP fields had to be removed. In both cases, necessary preprocessing required manual deletion of SNPs, for which the missing or invalid data could be restored, to allow MungeSumstats to proceed with restoration for the remainder of the test files. Additionally all non-traditionally formatted rsIDs e.g. “esv3584976”, were removed to prevent the automatic failure of the program.

### Assessment of SumStatsRehab and comparison with MungeSumstats

To assess the restoration of both tools, two different metrics were used. For qualitative attributes, accuracy was assessed in terms of concordance with the original summary statistics file. This was used for the chromosome, base pair location, effect allele, and other allele columns. For quantitative attributes, we calculated the difference between the predicted values and the original, masked values using Formula 1, which yields an accuracy score between 0 and 1. This was used to calculate the relative accuracy for the allele frequency, beta, standard error, and *p*-value columns, in order to account for floating point arithmetic and rounding errors.

1 $$1 - \min \left( {k\left| {x_{0} - x_{r} } \right|,1} \right)$$

where x_o_ and x_r_ are the original and restored values, and k is a fudge factor/an error term, which is different for each column. For allele frequency column: k = 2, for beta column: k = 6, for standard error column: k = 4, for *p*-value column: k = 3.

The overall accuracy for each column was calculated as the average of the accuracy metrics for each entry. These results were then used to assess and compare the restoration process of both SumStatsRehab and MungeSumstats.

We did not use any accuracy metrics with respect to evaluating restoration of rsIDs, as the rsID restoration is dependent on the publication timeframe of the GWAS. For earlier GWAS, rsID names do not correspond well to more current dbSNPs databases; the differences in rsID may not reflect differences in accuracy of restoration but differences in dbSNP versions.

## Results

### Restoration using SumStatsRehab

**Fig. 3:**
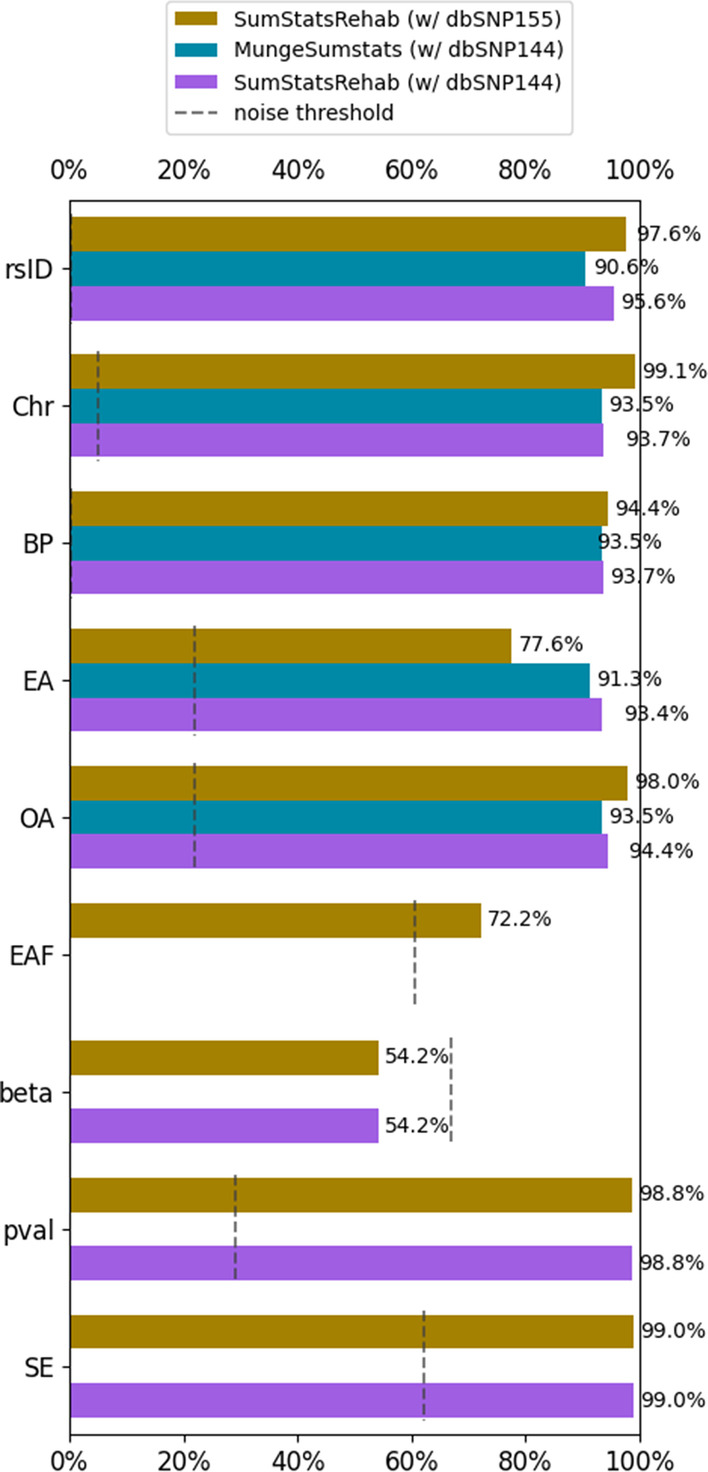
Comparison of average restoration accuracy using SumStatsRehab with dbSNP versions 144 and 155, as well as using MungeSumstats with dbSNP144. The noise threshold represents the expected level of accuracy achieved by restoring with a random value, and is intended to correct for correct restoration by chance

SumStatsRehab was able to successfully restore rsID, effect/other alleles, chromosome, base pair position, effect allele frequencies, beta, standard error, and *p*-values for all 27 test files. These restorations occurred without any SNP loss (Table 2). As the original c-reactive protein (CRP) file was missing variant ID data, Chr and BP restoration could not be assessed and was input as N/A. SumStatRehab managed to restore on average 97.6% and 95.6% of rsIDs accurately when using dbSNP155 and 144 respectively (Fig. 3). The discrepancy in rsID accuracy may be attributed to slight variations found in the earlier dbSNP dataset version used in the original GWAS’. Restoration accuracy was also greater than 93.7% for chromosome, base pair position, other allele, standard error, and *p*-values, while EA, EAF, and beta had restoration accuracies of 77.61%, 72.3% and 54.2%, respectively (Fig. 3). The quality of EA restoration using SumStatsRehab was largely dependent on the version of dbSNP used. When using dbSNP155, a newer version of the dbSNP database to restore older data, the greater number of alternate allele possibilities present in the newer database meant fewer direct restoration matches to the older data; however, when restoring EA data using the older dbSNP version 144, the number of direct matches was greatly increased to 93.4%. The reduced restoration accuracy of EAF is a function of population-dependent differences in EAF; restored EAFs are a naive approximation of the EAF as we don’t know the population-specific composition of the GWAS samples. Beta restoration at 54.2% can be attributed to unsigned standard error values, leading to inverse beta scores which prevented an exact match (https://github.com/Kukuster/SumStatsRehab#supplementary-information). Caution should be taken to only attempt beta restoration when signed standard error data is provided.

**Table 2:** Total % of SNPs removed per GWAS summary statistics file for restoration runs by SumStatsRehab and MungeSumstats

	MungeSumStats			SumStatsRehab		
	Allergies	Blood pressure	CRP	Allergies (%)	Blood pressure (%)	CRP
rsID	6.00%	12.05%	11.40%	0	0	0%
Chr	6.50%	Fail	N/A	0	0	N/A
BP	6.50%	Fail	N/A	0	0	N/A
EA	8.70%	Fail	Fail	0	0	0%
OA	6.50%	Fail	Fail	0	0	0%
MAF	N/A	N/A	N/A	0	0	0%
t-statistics	N/A	N/A	N/A	0	0	0%
*p*-value	N/A	N/A	N/A	0	0	0%
StdErr	N/A	N/A	N/A	0	0	0%

### Comparison of SumStatsRehab and MungeSumstats

As MungeSumstats is the only current alternative tool for data restoration, we also attempted restoration runs with MungeSumstats on the prepared test files. This comparison was performed using all 27 previously described test files derived from 3 GWAS, in order to compare the efficiency of restoring 9 data categories (Table 3). We assessed the comparison on the basis of restoration accuracy and data loss.

**Table 3:** Comparison of supported restoration categories for SumStatsRehab and MungeSumstats

Column restored	MungeSumstats	SumStatsRehab
rsID	Yes	Yes
Chr	Yes	Yes
BP	Yes	Yes
EA	Yes	Yes
OA	Yes	Yes
MAF	No	Yes
t-statistics	No	Yes
*p*-value	No	Yes
StdErr	No	Yes

MungeSumstats was initially unable to restore any of the test files associated with the blood pressure and CRP GWAS’. Despite significant preprocessing to prevent automatic failures, MungeSumstats still failed to restore all columns with the exception of rsID for these files (Table 2).

MungeSumstats removed 12.05% and 11.4% of SNPs, for the blood pressure and C-reactive protein files respectively, relative to the 0% loss achieved by SumStatsRehab (Table 2). Removed entries included rows where all entries were correct. For the allergy GWAS test files, MungeSumstats removed up to 8.7% of SNP entries, while restoring chromosome number, base pair position, rsID and allele columns. MungeSumstats does not restore missing allele frequencies, standard error, beta values and *p*-values; we were thus unable to compare our tool against MungeSumstats for these test cases. For all runs on the GWAS test files, SumStatsRehab had greater restoration accuracy than MungeSumstats (Fig. 3).

### Comparison of computational load

We also compared SumStatsRehab and MungeSumstats on the basis of execution time and memory usage. MungeSumstats had significantly lower run time for all restorations. When running tests sequentially or in parallel, the average execution time of MungeSumstats was around 6 min 53 s. Execution times for SumStatsRehab were significantly higher, with an average of 18 min 5 s, when running up to 6 tests in parallel, and 35 min when running all tests sequentially. The current implementation of SumStatsRehab runs processes sequentially, although its architecture leaves room for introducing parallelism in the future updates, while MungeSumstats architecture currently allows parallelization.

To compare memory usage, as SumStatsRehab can only be run sequentially, we chose to also run MungeSumstats sequentially to allow for equitable comparison. MungeSumstats used 12–16 GB of RAM during execution with rare drops into the 1–5 GB range, with a peak memory usage of 25 GB while unsuccessfully trying to restore the blood pressure GWAS effect allele test file. MungeSumstats’ system cache usage was 1–2 times the size of the input file, which varied between 0.5 and 1.5 GB. In contrast, SumStatsRehab used a maximum of 800 MB of RAM, and cache equivalent to the unpacked input file size.

## Discussion

A recent workshop set up to outline the best practice of sharing and standardizing GWAS summary statistics recommended that the following should be mandatory when sharing this data: a form of variant identifier, *p*-value, effect allele, other allele, effect allele frequency, effect and standard error [14]. While these recommendations will assist researchers going forward, there still remain thousands of incomplete GWAS summary statistics which may be able to provide insightful information for lesser studied health traits had they adhered to the aforementioned suggestions.

One potential avenue to address this issue lies in the restoration of incomplete columns of data. SumStatsRehab was able to restore this data in GWAS summary statistics, including the chromosome, base pair position, rsID, allele frequency, effect and other alleles, beta, *p*-value and standard error, more accurately and with less loss than any other currently available tool. These entries are key to generating robust polygenic risk score (PRS) models.

References to GWAS variants and their incorporation into PRS often relies upon correct identification of these variants by rsID [4, 15, 16]; SumStatsRehab is able to accurately restore rsID entries by overcoming several challenges associated with standardizing and inferring rsIDs. As SNP databases are updated, many rsIDs have been renamed across the different reference builds and versions, such that several different identifiers may refer to the same SNPs [17]. This may pose an issue when combining multiple GWAS which utilize different reference builds [17]. By rewriting rsID data using a specified reference database version, SumStatsRehab allows users to combine GWAS summary statistics which were generated using different databases. The ability to reliably combine GWAS as a result of proper rsID updating and restoration allows the identification of variants that were individually deemed insignificant in individual studies, but may play some role in disease or trait determination with increased power [18, 19].

Another key component of GWAS summary statistics is allele frequency data. One key use of this data is the identification of effect alleles [20]. The accurate restoration of missing minor allele frequencies poses a specific challenge, particularly when the ethnicity of the study cohort is not explicitly clear. The most straightforward method to restore this data, and the method implemented in SumStatsRehab, is to use allele frequencies contained within a genomic database such as dbSNP as a proxy, however genetic variability between the cohort of the target study and the data contained in these databases means that data restored this way will seldom provide an exact match to the original. As a result, users should be mindful of the limitations of EAF data restoration in downstream applications.

Beyond rsID and allele frequency, Standard error, *p*-values and beta are also important in the estimation of effect sizes when generating polygenic risk scores [15]. This data is often omitted from non-standardized publicly available GWAS summary statistic files, rendering the files unusable. To date, no tool currently allows for any of these three types of data to be restored. However, by utilizing the relationship between these three types of data [10], SumStatsRehab is able to closely predict the missing values, given that at least two of the three types of data are present. To our knowledge, SumStatsRehab is the only currently available tool which allows users to restore this type of data.

Before data can be restored, first the researcher must identify whether there are any issues with the dataset. While in most cases, incomplete identification and association data such as rsID or standard error may be obvious, this is not always the case. Manually identifying incomplete data entries can be a time-consuming and labor-intensive process, with GWAS summary statistic files now typically containing more than 8 million genetic variants [14]. The diagnose command implemented within the SumStatsRehab workflow provides visual aids, considerably increasing the speed at which issues with these files can be identified, and reducing the overall time spent processing data.

To our knowledge, SumStatsRehab represents the only tool which currently solely addresses data restoration to this extent. Alternative tools which also attempt partial data restoration, such as MungeSumstats, are primarily focused on data standardization. In order to assess the restoration quality of SumStatsRehab, we performed a comparison between this tool and MungeSumstats with regards to data restoration.

One of the major benefits of restoring non-standard GWAS summary statistic files using SumStatsRehab is the ability to specify column names using a JSON file input. This in turn allows users to utilize files with non-standardized layouts and headers. In comparison, MungeSumstats requires column headers to be in a specific format based on the standard input format of IEU GWAS VCF files. When this is not the case, it is unable to identify the effect allele column causing the process to fail.

One area in which SumStatsRehab underperformed was in the restoration of effect alleles when using dbSNP155. More recent dbSNP datasets contain additional alternative alleles, reducing the likelihood of an exact match between the newly restored file and the older original dataset which may have relied on an earlier dbSNP dataset release. MungeSumstats performed well when restoring EA data, however MungeSumstats relies on the most up-to-date SNPlocs dataset. These curated datasets inevitably lag behind the release of NCBI’s dbSNP datasets, and contain fewer SNPs. SumStatsRehab allows users to implement any build and version of the dbSNP database. So in instances such as this where restoration accuracy may be reduced when using newer dbSNP databases, users should take care to choose the most appropriate dbSNP build for their target data. In doing so, the restoration accuracy for effect alleles may be greatly increased. This is shown by contrasting the restoration accuracy for EA when using dbSNP144 compared with dbSNP155 in Fig. 3, with a 15.8% increase.

For all tested data categories, SumStatsRehab outperformed MungeSumstats in data restoration. However, it must be noted that if the researcher's goal is performing meta analyses or other types of analysis which require standardized data, MungeSumstats is a more appropriate tool.

## Conclusion

Overall, SumStatsRehab offers a highly maintainable tool which can easily be optimized for specific use cases with minimal modifications. This tool incorporates functional programming in addition to pipeline-like architecture, to define a flexible framework that is suitable for working on massive scales with various cloud computing platforms with minimal to no refactoring. The combined effect of this is a unique bioinformatics offering which allows users interested in generating PRS a method to increase the likelihood of being able to use GWAS summary statistics for any given health trait.

## Data Availability

The datasets generated and/or analyzed during the current study are available at the following web link: https://github.com/Kukuster/SumStatsRehab#supplementary-information.
